# Quantifying the Unseen Dengue Burden: A Municipality‐Level Ascertainment Surface for Pará State, Brazilian Amazon

**DOI:** 10.1002/jmv.71205

**Published:** 2026-11-05

**Authors:** Adriano Madureira dos Santos, Jean Arthur Costa Dias, Gui N. DeSouza, Karla Figueiredo, Marcos César da Rocha Seruffo

**Affiliations:** ^1^ Operational Research Laboratory (LPO) Federal University of Pará (UFPA) Belém Brazil; ^2^ Laboratory of Intelligent Interactive Systems (LIIS) Federal University of Pará (UFPA) Belém Brazil; ^3^ Vision‐Guided and Intelligent Robotics Lab (ViGIR), Electrical Engineering and Computer Science Department University of Missouri Columbia USA; ^4^ Department of Informatics and Computer Science, Institute of Mathematics and Statistics Rio de Janeiro State University (UERJ) Rio de Janeiro Brazil

**Keywords:** Bayesian spatiotemporal model, Brazilian Amazon, dengue fever, spatial ascertainment ratio, surveillance bias, under‐ascertainment

## Abstract

Passive dengue surveillance systematically underestimates true disease burden, and this under‐ascertainment is spatially structured rather than random. In the Brazilian Amazon, municipalities with the weakest notification infrastructure tend to coincide with those presenting the most favorable environmental conditions for vector proliferation. This work investigates whether municipalities with more active surveillance can serve as a structural proxy to infer cases in less active regions, allowing for the identification of localities where notification capacity fails to reflect true transmission. Building on this framework, this study aimed to estimate municipality‐level dengue under‐ascertainment in Pará by disentangling surveillance capacity from true transmission intensity through a Bayesian spatiotemporal model. A BYM2‐ST model was fitted to dengue notification data from municipalities of Pará, from January 2018 to December 2024, integrating socioeconomic indicators and time‐varying climatic covariates. A spatial ascertainment ratio was derived for each municipality from the model, benchmarking observed notifications against the structurally expected counterfactual. The results showed that only 20.1% of municipalities reported notification counts within the expected range, while 40.3% showed statistically supported evidence of under‐ascertainment.

## 1 Introduction

Dengue fever represents the most rapidly spreading arboviral disease worldwide, yet official statistics capture only a fraction of true infections because passive notification systems predominantly reflect local surveillance infrastructure rather than actual disease incidence [1, 2, 3]. This discrepancy between true burden and recorded incidence is particularly pronounced in Brazil, which reports the majority of dengue cases in the Americas [4]. During the record‐breaking outbreak in 2024, national emergency declarations highlighted how reporting completeness remains constrained by healthcare‐seeking behavior, diagnostic accuracy, and institutional infrastructure [5, 6, 7], patterns further disrupted by the COVID‐19 pandemic [8].

On a national scale, the Amazon region exhibits the highest environmental suitability for *Aedes* vector proliferation alongside severe fragmentation of health infrastructure [9, 10]. This tension is especially critical in the Brazilian state of Pará, where a complex territorial mosaic of urban centers, riverine communities, and forest‐edge settlements creates steep socioeconomic gradients [10]. In such low‐resource settings, passive notification systems only record individuals who access formal care, making under‐ascertainment bias a structural feature that renders the true disease burden systematically invisible where healthcare access is most constrained [11, 12, 13].

Because under‐capture is unevenly distributed across the territory, lower notification rates in under‐resourced municipalities often indicate detection deficits rather than reduced transmission intensity [14]. Allocating prevention resources based solely on crude reported cases risks directing investment toward populations with functional surveillance while leaving high‐risk, unobserved areas neglected [15].

To address this limitation, expected case modeling utilizes data from municipalities with active surveillance to inform expectations in infrastructure‐deficient regions [16]. Standard epidemiological modeling typically separates structural drivers into socioeconomic infrastructure [17] and climatic variables [18].

Existing analytical choices include global multiplication factors, which obscure the municipal heterogeneity necessary for targeted interventions [19], and seroprevalence surveys, whose high cost and cross‐sectional nature prevent continuous spatial extrapolation [20]. Without robust spatial resolution, ascertainment bias corrections remain insufficiently resolved for municipal surveillance planning.

Building on these considerations, the primary objective of this study is to map and quantify municipal notification bias using a spatial hierarchical framework. The analysis addresses three core questions: whether spatial notification patterns are primarily driven by surveillance capacity rather than transmission heterogeneity, whether high climatic suitability systematically co‐occurs with low notification capacity, and whether the resulting ascertainment bias surface remains stable across temporal disruptions to guide targeted surveillance investment.

## 2 Materials and Methods

This section details the study design, data sources, analytical model, and validation procedures that form the methodological framework for estimating municipality‐level dengue surveillance ascertainment across Pará. Figure 1 presents the schematic of the complete analytical workflow.

**Figure 1:**
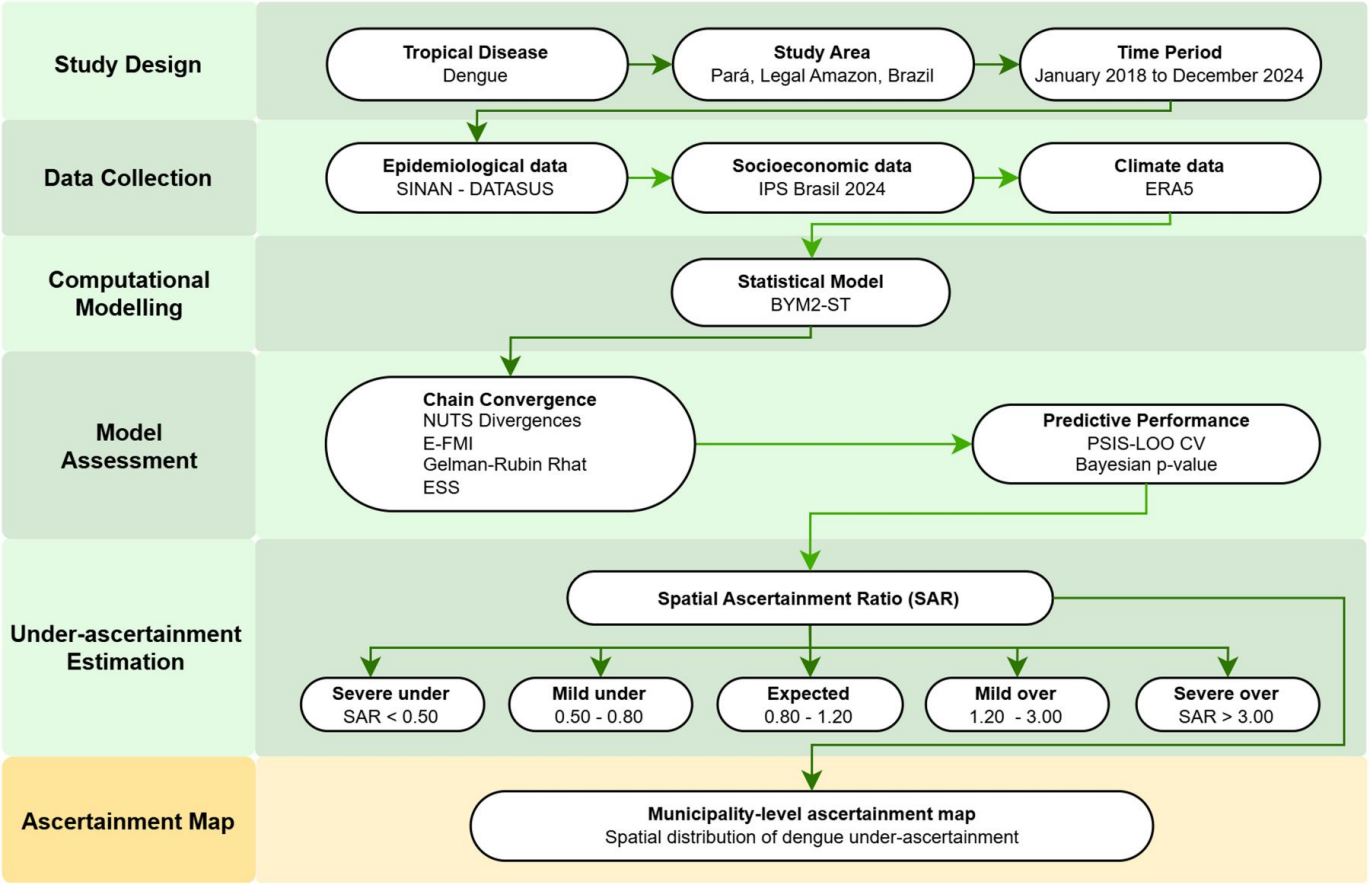
Methodological design of the work.

### 2.1 Study Design and Study Area

The operational rationale for this study focuses on evaluating municipal notification disparities where surveillance completeness is constrained by structural and geographic factors [21, 22]. In Brazil, while metropolitan health districts maintain integrated electronic reporting systems [23], remote Amazonian municipalities face significant logistical barriers and variable healthcare access that directly affect case detection [24]. Furthermore, because regional climatic conditions consistently favor *Aedes aegypti* proliferation across these remote territories [14, 25], the resulting notification records remain highly susceptible to spatial ascertainment bias.

Based on this analytical requirement, Pará was selected as the study setting to minimize confounding from disparate state‐level policies, funding allocations, and surveillance training programs [26]. Occupying a vast geographical extent as the second‐largest state in Brazil, its territorial structure combines a highly centralized health infrastructure in the Belém metropolitan area with severe logistical constraints in remote interior municipalities [9, 14]. This internal contrast establishes an ideal empirical framework to model how structural surveillance coverage shapes the spatial distribution of dengue notifications across time.

### 2.2 Epidemiological Data

Longitudinal dengue surveillance records were extracted from the Brazilian Information System for Notifiable Diseases (SINAN), maintained by the Ministry of Health through DATASUS infrastructure under the national compulsory notification framework [27, 28, 29]. This database records all clinically suspected and laboratory‐confirmed cases reported by public and private health facilities across the national territory [28]. The raw monthly counts were aggregated at the municipal level for all 144 municipalities of Pará, establishing a uniform unit of analysis for evaluating spatial and temporal notification patterns across the entire state surface over time.

The observational window spans the period from January 2018 through December 2024, yielding a continuous sequence of 84 calendar months. This 84‐month timeframe captures distinct epidemiological dynamics, including the pre‐pandemic baseline, the operational disruptions to healthcare seeking and surveillance during the COVID‐19 pandemic, and the unprecedented national dengue surge in 2024 [8, 30, 31]. By spanning these contrasting transmission regimes, the temporal design ensures sufficient longitudinal variability to robustly estimate structured temporal random effects and evaluate the stability of notification capacity across epidemic shocks.

### 2.3 Covariates

Municipality‐level ascertainment bias estimation relies on predictors capturing both structural features and dynamic environmental conditions across the territory. In this sense, two complementary covariate sets were assembled for the 144 municipalities of Pará, incorporating 26 structural indicators from IPS Brasil [32] and 3 spatiotemporal climatic variables derived from ERA5‐Land reanalysis [33]. The IPS Brasil indicators operationalize key social, economic, and institutional determinants of dengue notification completeness identified in the literature [11, 17, 20, 21]. Concurrently, monthly aggregations of temperature, precipitation, and relative humidity extracted within municipal boundaries provide proximate indicators of *Aedes aegypti* ecology and seasonal transmission suitability [16, 25, 34]. Full specifications for the complete set of 29 predictors are detailed in Supplementary Material S1, with individual indicators cataloged in Table S1.

Prior to model inclusion, an outcome‐agnostic three‐stage verification comprising spatial dispersion, pairwise Pearson correlation, and Variance Inflation Factors assessed the geometric properties of the predictor matrix across municipalities. Low spatial dispersion was identified in temperature, relative humidity, and life expectancy, yet these variables were retained given their fundamental biological and epidemiological role in dengue transmission dynamics. Diagnostic evaluation flagged strong pairwise correlation between precipitation and relative humidity, alongside moderate collinearity among select health and security indicators. However, iterative VIF testing converged without removals, yielding a maximum VIF of 8.50 for relative humidity, well below the conservative threshold of 10.0. Consequently, all 29 predictors were confirmed statistically suitable and retained for the BYM2‐ST framework.

### 2.4 Statistical Model

Dengue notification counts derived from passive surveillance systems exhibit overdispersion, spatial autocorrelation, and marked spatiotemporal dynamics characterized by seasonal peaks and interannual epidemic surges [16, 25]. Addressing these intrinsic stochastic dependencies, a Bayesian hierarchical spatiotemporal model was formulated [35, 36]. For municipality i at month t, the observed notification count $Y_{it}$ has conditional expectation $\mu_{it}$, linked to municipal covariates and random effects through the linear predictor defined in Equation (1).

(1) $$\log\left(\mu_{it}\right)=\log\;\left(\frac{P_{i}}{12}\right)+\alpha +X_{it}^{⊤}\beta +\varphi_{i}+\tau_{y\left[t\right]}+\delta_{s\left[t\right]},$$

In Equation (1), $P_{i}$ represents the municipal census population adjusted to a monthly scale as an offset, α denotes the baseline intercept, and $\beta \in R^{29}$ captures the regression coefficients associated with the 26 IPS Brasil indicators and 3 ERA5‐Land climatic predictors in $X_{it}$. The terms $\tau_{y\left[t\right]}$ and $\delta_{s\left[t\right]}$ specify the structured annual trend and calendar‐month seasonality [2, 10, 14]. Spatial heterogeneity is modeled through the scaled BYM2 spatial random effect $\varphi_{i}$ [37], formally presented in Equation (2).

(2) $$\varphi_{i}=\sigma_{s}\;\left[\sqrt{\rho_{s}}\;\frac{{\tilde{\varphi }}_{i}^{\text{ICAR}}}{\sqrt{\tau }}+\sqrt{1-\rho_{s}}\varepsilon_{i}\right],$$

In Equation (2), $\sigma_{s}>0$ controls the total spatial standard deviation, $\rho_{s}\in \left(0,1\right)$ governs the mixing proportion between spatially structured and unstructured components, τ represents the graph‐theoretic scaling factor for the intrinsic autoregressive prior [38], and $\varepsilon_{i}$ accounts for independent municipal noise. Conditional on covariates and temporal effects, $\varphi_{i}$ isolates the municipal spatial ascertainment effect required to evaluate notification bias.

### 2.5 Model Assessment

Bayesian MCMC convergence and Markov chain sampling efficiency were assessed across the joint posterior sample using standard diagnostic metrics [39, 40]. Specifically, evaluation included NUTS divergent transitions, the energy fraction of missing information, the Gelman–Rubin convergence statistic $\hat{R}$ with a convergence threshold below 1.01, and the minimum bulk effective sample size for regression parameters. Model predictive accuracy was evaluated using Pareto‐Smoothed Importance Sampling Leave‐One‐Out cross‐validation [41]. Reliability of the cross‐validation estimates was monitored through Pareto k diagnostic shape values [42], while posterior predictive checks, presented in Equation (3), evaluated global fit by comparing observed outcome statistics against replicates drawn from the joint posterior distribution.

(3) T(y)=Pr(y=0)

Equation (3) defines the primary discrepancy statistic as the empirical proportion of zero‐notification municipality–month cells across the study region. Posterior predictive calibration was formally evaluated through the Bayesian p‐value, calculated as the tail probability that replicated statistics exceed observed values [43]. Values positioned within the conservative interval between 0.05 and 0.95 indicate acceptable model calibration without systematic overdispersion or zero‐inflation bias.

### 2.6 Under‐Ascertainment Estimation

The municipality‐specific spatial random effect $\varphi_{i}$ recovered by the BYM2 model captures systematic deviations between observed notification counts and baseline predictions derived from municipal covariates and temporal dynamics. Within this framework, this spatial residual is interpreted as a municipality‐level ascertainment index. The spatial ascertainment ratio (SAR) is defined as the exponentiated posterior mean of $\varphi_{i}$, formally expressed in Equation (4).

(4) $${\text{SAR}}_{i}=\text{exp}\left(\varphi_{i}\right).$$

A SAR value below unity identifies municipal under‐ascertainment due to surveillance gaps exceeding covariate‐predicted expectations, whereas values above unity reflect over‐ascertainment driven by active case‐finding or heightened reporting effort. Conversely, values approaching unity reflect notification rates fully aligned with baseline expectations. Based on this formulation, municipalities were stratified into five ordinal categories detailed in Table 1, adapting continuous ratio visualization principles established in the literature [44].

**Table 1:** SAR classification scheme and threshold definitions.

Category	SAR range
Severe under‐ascertainment	SAR<0.50
Mild under‐ascertainment	0.50≤SAR<0.80
Expected ascertainment	0.80≤SAR<1.25
Mild over‐ascertainment	1.25≤SAR<3.00
Severe over‐ascertainment	SAR≥3.00

## 3 Results

This section is organized into two subsections: the first reports the estimated model parameters and covariate associations, while the second presents the spatial ascertainment mapping. Detailed experimental setup specifications are provided in Supplementary Material S1.

### 3.1 Model Parameters and Covariate Effects

Rigorous evaluation of MCMC convergence and BYM2‐ST fit confirmed that the Markov chain Monte Carlo sampling achieved numerical stability and calibration across the 12,096 municipality–month observations [40, 45]. Table 2 details that sampling convergence metrics alongside predictive cross‐validation and Bayesian p‐value calibration demonstrated optimal performance without zero‐inflation bias [41, 43]. Consequently, the estimated spatial, temporal, and dispersion hyperparameters provided a statistically sound basis for epidemiological inference regarding disease ascertainment patterns.

**Table 2:** Posterior sampling diagnostics, model fit metrics, and key hyperparameters for BYM2‐ST model.

Metric/parameter	Estimate/value	Reference/94% HDI
Sampling convergence		
Divergent transitions	0	=0
Energy FMI (E‐FMI)	0.854	>0.20
Maximum $\hat{R}$ (all params.)	1.003	<1.01
Model fit & calibration		
Pareto $\hat{k}<0.50$	12,059/12,096 (99.7%)	>90%
Observed P(y=0)	56.3%	—
Predicted P(y=0)	55.8%	54.7–56.8
Bayesian p‐value	0.156	0.05–0.95
Model parameters & hyperparameters		
Marginal spatial SD $\sigma_{s}$	1.488	1.116–1.867
Spatial mixing proportion $\rho_{s}$	0.604	0.106–1.000
Interannual SD $\sigma_{\text{year}}$	0.658	0.375–0.987
Seasonal SD $\sigma_{\text{sazon}}$	0.471	0.287–0.677
Negative binomial dispersion $\alpha_{\text{nb}}$	0.378	0.364–0.392

Epidemiological inference from the posterior parameter estimates signals marked structural heterogeneity and dynamic temporal components across the territory. The magnitude of the spatial variance suggests substantial unexplained geographical variation in notification compliance, with approximately 60% of this spatial structure originating from spatial clustering among neighboring municipalities and 40% from local municipal variability. Furthermore, the interannual and seasonal variability components estimate strong macro‐level temporal fluctuations and marked within‐year transmission cycles, while the estimated Negative Binomial dispersion parameter points to substantial extra‐Poisson variability in the observed notification counts across the study period.

Temporal random effects displayed in Figure 2 illustrate pronounced within‐year seasonality alongside substantial interannual variability across the historical series. The seasonal signal highlights an epidemic transmission window concentrated in the first 4 months of the year, with peak transmission occurring between February and April. Conversely, the final quarter from September to December represents the seasonal trough characterized by sustained transmission reduction. The statistical support for this intra‐annual dynamic is demonstrated by the majority of calendar months displaying posterior intervals excluding zero, reflecting a robust and regular seasonal baseline across Pará.

**Figure 2:**
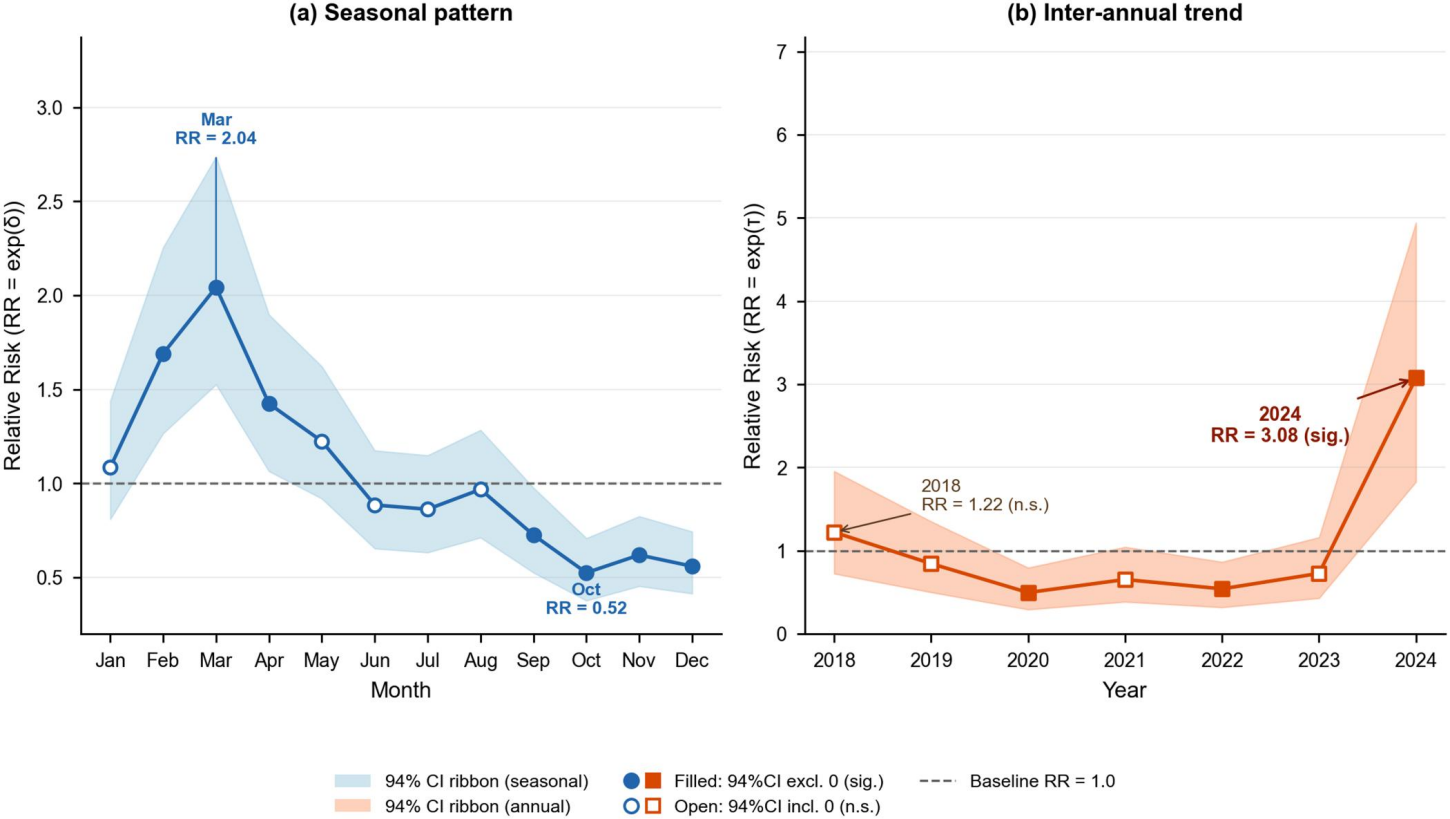
Posterior mean temporal random effects and 94% highest density intervals.

Complementing the seasonal baseline, the estimated interannual trajectory captures distinct periods of suppressed epidemic activity contrasted with sharp disease resurgence. Substantial transmission reductions coincided temporally with the healthcare disruptions of the COVID‐19 pandemic in 2020 and 2022, during which notification rates fell to roughly half the long‐term historical mean. In contrast, transmission intensity peaked sharply in 2024, exhibiting a threefold surge above baseline levels that directly aligns with the national dengue emergency. These combined dynamics confirm that passive notification patterns are shaped by strong seasonal forcing superimposed on macro‐level epidemic cycles.

The 29 candidate predictors contribute to the municipality‐specific estimated expected count through their posterior mean estimates. This constitutes the structural layer used to characterize dengue notification heterogeneity across Pará, with posterior incidence rate ratios and 94% HDIs presented in Table 3. Only the climatic predictors yielded HDIs fully excluding zero, indicating that climate is the sole domain with a statistically credible directional association. This is consistent with the role of standing water in supporting *Aedes aegypti* breeding site expansion.

**Table 3:** Posterior incidence rate ratios for all 29 model covariates.

Variable	IRR (94% HDI)
Climate	
Monthly mean relative humidity (%)	1.447 (1.309, 1.598)*
Monthly total precipitation (mm)	1.088 (1.016, 1.168)*
Monthly mean temperature (°C)	1.075 (0.944, 1.232)
Sanitation	
Adequate solid waste collection	1.355 (0.962, 1.881)
Adequate sewage access	0.941 (0.767, 1.159)
Health	
Adult mortality rate (15–50 years)	1.463 (0.994, 2.184)
Non‐communicable disease mortality	1.163 (0.807, 1.714)
Suicide rate	1.112 (0.865, 1.411)
Life expectancy at birth	1.039 (0.814, 1.342)
ACSC hospitalization rate	0.987 (0.794, 1.222)
Infant mortality rate (under 5)	0.954 (0.761, 1.215)
ACSC‐adjusted mortality rate	0.938 (0.674, 1.275)
Environment	
${\;\text{CO}}_{2}$ emissions per capita	1.305 (0.906, 1.866)
Vegetation suppression rate	1.067 (0.764, 1.515)
Urban green areas	1.031 (0.839, 1.280)
Heat foci density	0.946 (0.679, 1.328)
Security	
Homicide rate	1.048 (0.739, 1.473)
Education	
Secondary school dropout rate	1.135 (0.891, 1.445)
Primary school dropout rate	0.924 (0.725, 1.166)
Urbanization	
Employees with higher education	1.210 (0.961, 1.544)
Adequate housing floors	1.152 (0.874, 1.504)
Fixed broadband density	1.013 (0.768, 1.327)
Urban parks and plazas	0.931 (0.765, 1.139)
Cultural and leisure access	0.930 (0.730, 1.184)
Adequate housing walls	0.846 (0.627, 1.155)
Mobile telephony density	0.810 (0.568, 1.215)
Water	
Water distribution loss index	1.097 (0.875, 1.364)
Piped water access rate	0.960 (0.714, 1.267)
Water supply coverage index	0.901 (0.693, 1.189)

*94% HDI excludes the null value of 1.00.

The remaining predictors not achieving 94% HDI exclusion of zero do not imply their absence of contribution to the municipality‐specific expected count. Rather, their wide HDIs reflect that the available panel lacks sufficient statistical resolution to reliably establish each structural predictor's directional sign independently of the others, so that the heterogeneity they leave unaccounted for tends to accumulate in the spatial random effect $\varphi_{i}$, supporting its interpretation as a conservative index of residual ascertainment deviation [35].

Finally, the BYM2‐ST posterior suggests that notification heterogeneity across Pará's 144 municipalities is both large and geographically structured. The covariate layer identifies climatic humidity and precipitation as the only predictors with individually credible directional associations. The remaining predictors contribute collectively through individually unresolved directional effects across the 144‐municipality landscape. The heterogeneity that covariates and temporal effects cannot account for is captured by the spatial random effect $\varphi_{i}$, whose exponential transform constitutes the spatial ascertainment ratio examined in the following subsection.

### 3.2 Spatial Ascertainment Mapping

The BYM2‐ST posterior characterizes substantial residual notification heterogeneity across Pará's 144 municipalities and the majority of the residual is geographically structured along the contiguity graph. In this context, Figure 3 displays the geographic distribution of this estimated residual heterogeneity, representing the estimated municipality‐specific SAR for all Pará municipalities across the 2018–2024 panel.

**Figure 3:**
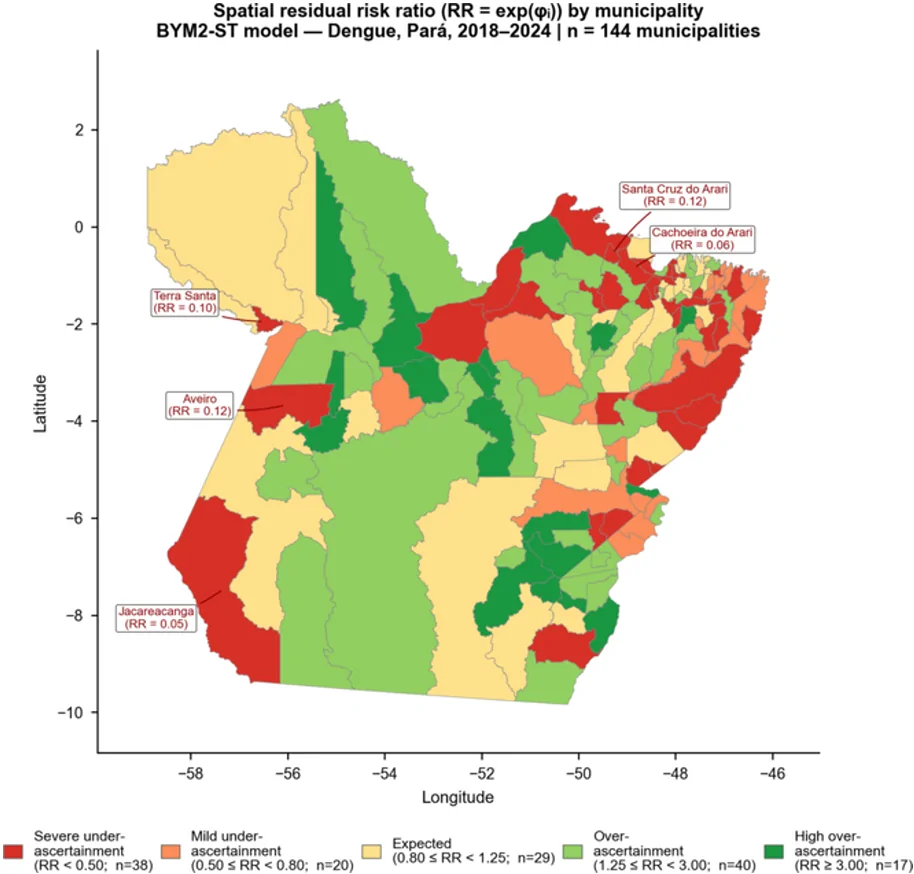
Spatial ascertainment ratio.

Across the state, under‐ascertainment reached approximately 40.3% of the municipalities, with 65.5% of these meeting the threshold for severe under‐ascertainment, whereas over‐ascertainment was identified in 39.6% of the territory. The spatial analysis reveals two contiguous and geographically coherent clusters of severe under‐ascertainment. The first cluster comprises the southwestern and western territory, spanning the Tapajós basin and the southern Baixo Amazonas corridor, where remote access and logistically challenging transport severely limit local disease reporting. The second cluster occupies the Marajó Archipelago in northeastern Pará, a region historically marked by structural socioeconomic vulnerabilities, sanitation deficits, and limited healthcare coverage that directly hinder epidemiological surveillance [46]. Table 4 summarizes the ten municipalities with the most pronounced notification deficits based on their SAR values and 94% HDIs.

**Table 4:** Ten municipalities with the most severe under‐ascertainment.

Municipality	SAR	$\varphi_{i}$ 94% HDI
Jacareacanga	0.054	(−4.88,−0.99)
Cachoeira do Arari	0.055	(−4.29,−1.63)
Terra Santa	0.102	(−3.50,−1.11)
Aveiro	0.115	(−3.72,−0.71)
Santa Cruz do Arari	0.117	(−3.87,−0.52)
Cachoeira do Piriá	0.140	(−2.95,−0.99)
Gurupá	0.176	(−2.84,−0.65)
Ourém	0.182	(−2.88,−0.55)
Porto de Moz	0.196	(−2.91,−0.40)
Curionópolis	0.217	(−2.51,−0.53)

In sharp contrast to these under‐reported regions, over‐ascertainment concentrated along the eastern and southeastern corridor of Pará, specifically within the Araguaia‐Tocantins basin and neighboring border regions. Within this zone, SAR values consistently surpassed the 1.25 threshold, depicting an empirical spatial pattern that remains uninvestigated further due to the inherent limitations of notification data in resolving underlying structural drivers.

Humidity and precipitation are consistent with established drivers of dengue transmission and raise the model's expected notification count $\mu_{it}$ through their time‐varying effects. At the cross‐sectional level, however, their mean annual values show a negative association with municipality‐level estimated SAR. This pattern suggests that the most climatically at‐risk municipalities may also be the most severely under‐ascertained relative to their structural predictor profile. Specifically, municipalities with higher mean annual relative humidity yield systematically lower estimated SAR values, with an estimated Spearman rank correlation ρ of −0.240 and an estimated p‐value of 0.004. The same direction holds for the mean annual total precipitation, yielding an estimated ρ of −0.216 and an estimated p‐value of 0.009, as displayed in Figure 4. This persistent pattern is consistent with a structural climate‐surveillance gap rather than a transient, seasonal association.

**Figure 4:**
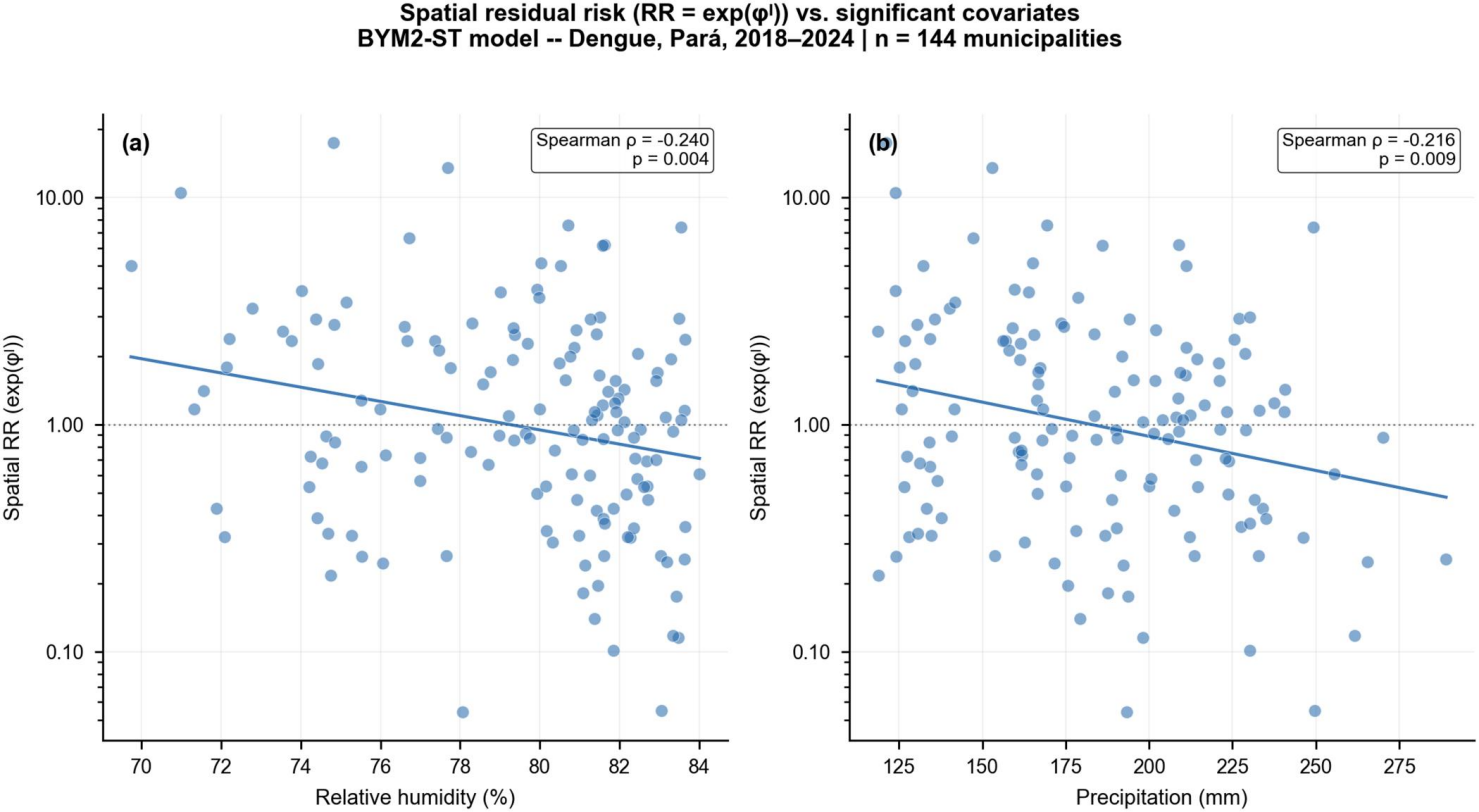
Spatial residual risk versus significant covariates: (a) Municipality‐level spatial ascertainment ratio versus mean annual relative humidity; (b) Mean annual total precipitation.

The two severe under‐ascertainment zones, western Pará and the Marajó Archipelago, are also characterized by the most humid and wet environments in the state. This co‐location suggests a compound surveillance deficit that passive notification data alone cannot resolve. The spatial autocorrelation carries practical implications for surveillance policy. The estimated ascertainment capacity suggests a tendency to concentrate geographically. This spatial concentration is consistent with socioeconomic and healthcare‐related patterns that may propagate surveillance quality across neighboring units. A targeted intervention in multiple severely under‐ascertained municipalities could potentially support positive spillovers into adjacent municipalities, although this hypothesis would require empirical validation.

The widespread occurrence of under‐ascertainment suggests that dengue surveillance performance varies substantially across municipalities. Because these residual deficits persist after adjustment for climatic, socioeconomic, demographic, and healthcare‐related factors, observed notification counts alone are consistent with a distorted representation of the true dengue burden across Pará. Spatial adjustment is therefore supported as a critical step for identifying municipalities where surveillance limitations are most likely to conceal underlying transmission.

## 4 Discussion

The empirical findings from the BYM2‐ST model confirm that dengue notification heterogeneity across Pará reflects structural asymmetries in local surveillance capacity rather than genuine variations in disease transmission alone. While the credibility of this diagnostic surface rests on the Bayesian hierarchical framework, its posterior predictive performance, and its alignment with established literature, this spatial structure effectively separates unobserved institutional capacity from underlying biological risk factors.

Addressing the spatial drivers of this burden, the scale of ascertainment failure across the state is fundamentally tied to structural discrepancies in municipal reporting profiles. The vast majority of municipalities deviate significantly from expected notification rates, indicating that raw case counts systematically misrepresent disease burden [21, 47]. Crucially, humidity and precipitation emerged as the key climatic drivers [48, 49], revealing a critical spatial inversion where regions with the highest environmental suitability for *Aedes aegypti* proliferation exhibit the most severe institutional suppression of formal notifications. Consequently, allocation strategies based strictly on crude SINAN records will systematically direct resources toward better‐served populations while bypassing those facing the highest unobserved transmission risk.

Translating these spatial patterns into planning tools, the estimated SAR surface provides a stable, spatially resolved ranking of surveillance adequacy that remains reliable even during epidemic surges [5]. Because the seasonal hydrological cycle is absorbed by the temporal components of the model [50], the resulting spatial residuals isolate chronic structural gaps rather than transient seasonal fluctuations [14]. This temporal stability enables regional health authorities, such as the *Programa Nacional de Controle da Dengue* (PNCD) and the *Secretaria de Estado de Saúde Pública do Pará* (SESPA), to utilize the index for long‐term resource allocation across *Superintendências Regionais de Saúde*.

Collectively, these findings demonstrate that spatiotemporal hierarchical modeling successfully leverages observed data from active surveillance municipalities to establish baseline expectations for lower‐capacity regions. By quantifying where the formal epidemiological record diverges most severely from true disease burden, this approach delivers a robust, actionable framework for targeted public health intervention.

## 5 Conclusion

This study applied a BYM2‐ST model to dengue notification data from Pará municipalities over 7 years, integrating structural and climate predictors to derive a municipality‐level SAR surface. Three findings emerged: (i) notification heterogeneity across the state proved predominantly structural; (ii) the zones most in need of a structural correction were those with the highest climatic transmission suitability; and (iii) the SAR surface remained stable across the collapse periods, confirming reliability for active case search. These findings reflect the resolution at which publicly available Brazilian surveillance data operate.

Multi‐state replication across Pará's Amazonian neighbors would establish whether the structural inversion between notification capacity and climatic transmission suitability extends beyond state boundaries. Prospectively, the posterior SAR distribution estimated here constitutes an informative prior for a continuously updated monitoring system in which incoming SINAN data would refine the ascertainment map across future epidemic cycles.

## Author Contributions

**Adriano Madureira dos Santos:** conceptualization, methodology, software, validation, formal analysis, investigation, data curation, writing – original draft, writing – review and editing, visualization, project administration. **Jean Arthur Costa Dias:** conceptualization, formal analysis, investigation, visualization, writing – original draft, writing – review and editing. **Gui N. DeSouza:** conceptualization, formal analysis, investigation, writing – original draft, writing – review and editing, project administration. **Karla Figueiredo:** conceptualization, formal analysis, investigation, writing – original draft, writing – review and editing, project administration. **Marcos César da Rocha Seruffo:** conceptualization, formal analysis, investigation, writing – original draft, writing – review and editing, project administration.

## Ethics Statement

Not applicable. This study analyzed secondary, anonymized epidemiological surveillance data, requiring no direct involvement or interaction with human subjects or animals.

## Conflicts of Interest

The authors declare that they have no known competing financial interests or personal relationships that could have appeared to influence the work reported in this paper.

## Supporting information


Supporting File

## Data Availability

The epidemiological datasets analyzed during the current study are publicly available from the Brazilian Ministry of Health through the DATASUS/SINAN platform (https://datasus.saude.gov.br). The demographic and territorial boundary data are openly accessible from the Brazilian Institute of Geography and Statistics (IBGE, https://www.ibge.gov.br). The socioeconomic covariates were obtained from the Social Progress Index Brazil (IPS Brasil, https://ipsbrasil.org.br). Any code or processed data used in this study are available from the corresponding author upon reasonable request.
