# Supplementary material for: Quantifying the Unseen Dengue Burden: A Municipality‐Level Ascertainment Surface for Pará State, Brazilian Amazon
Source: J Med Virol. 2026 Nov 5;98(11):e71205. doi: 10.1002/jmv.71205 (PMC13739229; doi:10.1002/jmv.71205)
Supplement: Supplementary file 1 [file JMV-98-e71205-s001.pdf]

Supplementary Material S1

Experimental Setup

Complement to the manuscript:

Quantifying the unseen dengue burden: a municipality-level ascertainment surface for Pará State, Brazilian Amazon

1 | COVARIATE SELECTION

The complete catalog of the 29 municipal-level predictors, including raw sources, spatial metrics, and individual Variance Inflation Factors (VIF_j), is detailed in Table S1. These covariates were submitted to a three-stage structural verification to assess their suitability for inclusion in the hierarchical BYM2 space-time model.

This diagnostic process was designed as a baseline verification to map the structural landscape of data. This verification was deliberately designed to operate independently of the disease outcome, evaluating only the geometric and algebraic properties of the predictor matrix across the 144 municipalities. Such outcome-agnostic screening avoids circular reasoning and ensures that covariate selection reflects structural adequacy. The verification comprised three complementary stages. First, we computed the spatial coefficient of variation CV_j for each covariate across municipalities, defined as presented in Equation S1.

$$CV_j = \frac{\sigma_j}{|\mu_j|}, \qquad j = 1, \ldots, 29, \tag{S1}$$

In Equation S1, μ_j and σ_j denote the mean and standard deviation of covariate j over the 144 municipalities. Variables with $CV_j < 0.10$ were flagged as exhibiting low spatial dispersion. The monthly mean temperature, life expectancy at birth, and monthly mean relative humidity covariates exhibited low spatial variation, with computed spatial coefficient of variation values of 0.014, 0.018, and 0.044, respectively, which were below the 0.10 threshold. Despite their low spatial variation, these variables were retained due to their well-documented theoretical relevance to dengue transmission and socioeconomic vulnerability. Second, we examined all pairwise Pearson correlation coefficients r_{jk} to identify potential collinearity through Equation S2.

$$r_{jk} = \frac{\sum_{i=1}^{N}(x_{ij} - \bar{x}_j)(x_{ik} - \bar{x}_k)}{\sqrt{\sum_{i=1}^{N}(x_{ij} - \bar{x}_j)^2}\sqrt{\sum_{i=1}^{N}(x_{ik} - \bar{x}_k)^2}}, \quad 1 \leq j < k \leq 29, \tag{S2}$$

TABLE S1 Covariates included in the spatiotemporal model.

Variable	Domain
Adequate sewage access*	Sanitation
Adequate solid waste collection*	Sanitation
Water supply coverage index*	Water
Piped water access rate*	Water
Water distribution loss index*	Water
Heat foci density*	Environment
CO_2e emissions per capita*	Environment
Vegetation suppression rate*	Environment
Urban green areas*	Environment
Adequate housing walls*	Sanitation
Adequate housing floors*	Sanitation
Urban parks and plazas*	Urbanisation
Fixed broadband density*	Urbanisation
Mobile telephony density*	Urbanisation
Employees with higher education*	Urbanisation
Cultural and leisure access*	Urbanisation
ACSC hospitalisation rate*	Health
ACSC adjusted mortality rate*	Health
Infant mortality rate (under 5)*	Health
Adult mortality rate (15–50 years)*	Health
Non-communicable disease mortality*	Health
Life expectancy at birth*	Health
Suicide rate*	Health
Primary school dropout rate*	Education
Secondary school dropout rate*	Education
Homicide rate*	Security
Monthly mean temperature (°C)**	Climate
Monthly total precipitation (mm)**	Climate
Monthly mean relative humidity (%)**	Climate

*Derived from IPS Brasil dataset [1].

**Derived from ERA5-Land reanalysis [2]

Equation S2 allows to flag pairs with $|r_{jk}| > 0.70$ for theoretical adjudication, where x_{ij} represents the value of covariate j in

municipality i. The strongest association was observed with an r value of 0.87 between monthly total precipitation and monthly mean relative humidity. Three other pairs also exceeded the threshold—namely, adult mortality rate and homicide rate, CO_2 emissions per capita and heat foci density, and ACSC-adjusted mortality rate and non-communicable disease mortality—with r values of 0.75, 0.73, and 0.71, respectively. These flagged pairs were subjected to formal multicollinearity diagnostics in the subsequent step, treating collinearity as a condition to be monitored rather than an automatic ground for removal. Third, we estimated Variance Inflation Factors (VIF) iteratively for each predictor j through Equation S3.

$$\mathrm{VIF}_j = \frac{1}{1 - R_j^2}, \tag{S3}$$

In Equation S3, R_j^2 is the coefficient of determination obtained by regressing X_j against all remaining 28 predictors. At each iteration, the variable with the largest VIF was targeted for removal if it exceeded the conservative threshold of VIF > 10.0, provided that such extreme collinearity was accompanied by compromised diagnostics in the previous stages and lacked overriding theoretical necessity. The iterative procedure converged in a single step with zero removals, as all covariates satisfied the $\mathrm{VIF}_j \leq 10.0$ criterion. The maximum VIF obtained was 8.50 for monthly mean relative humidity, confirming that the correlation identified in the second stage did not translate into critical multicollinearity. Consequently, all 29 covariates were deemed statistically adequate and retained for the BYM2-ST model.

2 | EXPERIMENTAL SETUP

Reliable estimation from the Pará dengue surveillance panel required analytical decisions calibrated to its distributional and spatial characteristics. Each decision directly determined the posterior and, consequently, every estimate reported below. This subsection documents those decisions together with the empirical evidence confirming that each was appropriate for this surveillance panel.

The analysis covered all 144 municipalities of Pará state, which belongs to the Brazilian Legal Amazon, from January 2018 to December 2024, comprising 84 monthly time points and 12,096 municipality–month observations. Over this period, 67,206 dengue notifications were recorded in SINAN, corresponding to a mean annual notification rate of approximately 111 cases per 100,000 inhabitants across the state. However, this state-level figure conceals substantial heterogeneity in the distribution of notifications. A total of 56.3% of all municipality–month cells registered zero notifications, reflecting both low transmission outside epidemic peaks and possible heterogeneity in case detection and reporting. As shown in Figure S1, the cumulative notification rate varied markedly across municipalities, illustrating the spatial heterogeneity that motivates the ascertainment correction.

The distributional form for the notification counts was selected empirically rather than assumed a priori. The raw counts showed strong overdispersion, with the monthly variance far exceeding the mean across municipalities. A Negative Binomial likelihood was therefore adopted[3]. The posterior predictive checking supported this likelihood choice as appropriate. A Zero-Inflated Negative Binomial extension was not required. The posterior predictive diagnostics suggested that the Negative Binomial BYM2 specification adequately reproduced the observed proportion of zero counts, indicating that additional zero-inflation parameters were unnecessary for this dataset. Prior distributions were assigned to reflect weakly informative beliefs calibrated to the natural scales of each parameter, supporting computational stability without imposing restrictive constraints on the posterior.

Although Penalized Complexity (PC) priors are commonly recommended for the spatial components of the BYM2 formulation, weakly informative standard Half-Normal priors were adopted here for the standard deviation parameters. Given the log-link function of the Negative Binomial model, this weakly informative regularizing prior effectively restricts epidemiologically implausible extreme variations while remaining non-informative over the plausible range of regional heterogeneity. This choice yielded high computational efficiency and excellent chain convergence under the NUTS sampler. These joint prior assignments are summarized in Table S2.

TABLE S2 Prior distributions assigned to all model parameters.

Parameter	Prior
Global Intercept – α	Normal(0, 2)
Covariates – β_k, $k = 1, \ldots, 29$	Normal(0, 1)
BYM2 Marginal SD – σ_s	HalfNormal(1)
ICAR Mixing Proportion – ρ_s	Beta(0.5, 0.5)
Interannual SD – σ_{year}	HalfNormal(0.5)
Seasonal SD – σ_{sazon}	HalfNormal(0.5)
NB Dispersion – α_{nb}	HalfNormal(1)

The global intercept α was assigned a weakly informative prior on the log-rate scale, which supports the estimated baseline notification level to be informed primarily by the data. The 29 regression coefficients β_k received regularization priors designed to guide the estimated incidence rate ratios toward a plausible range for each covariate. This estimated range is sufficiently flexible to accommodate potential dengue risk-factor

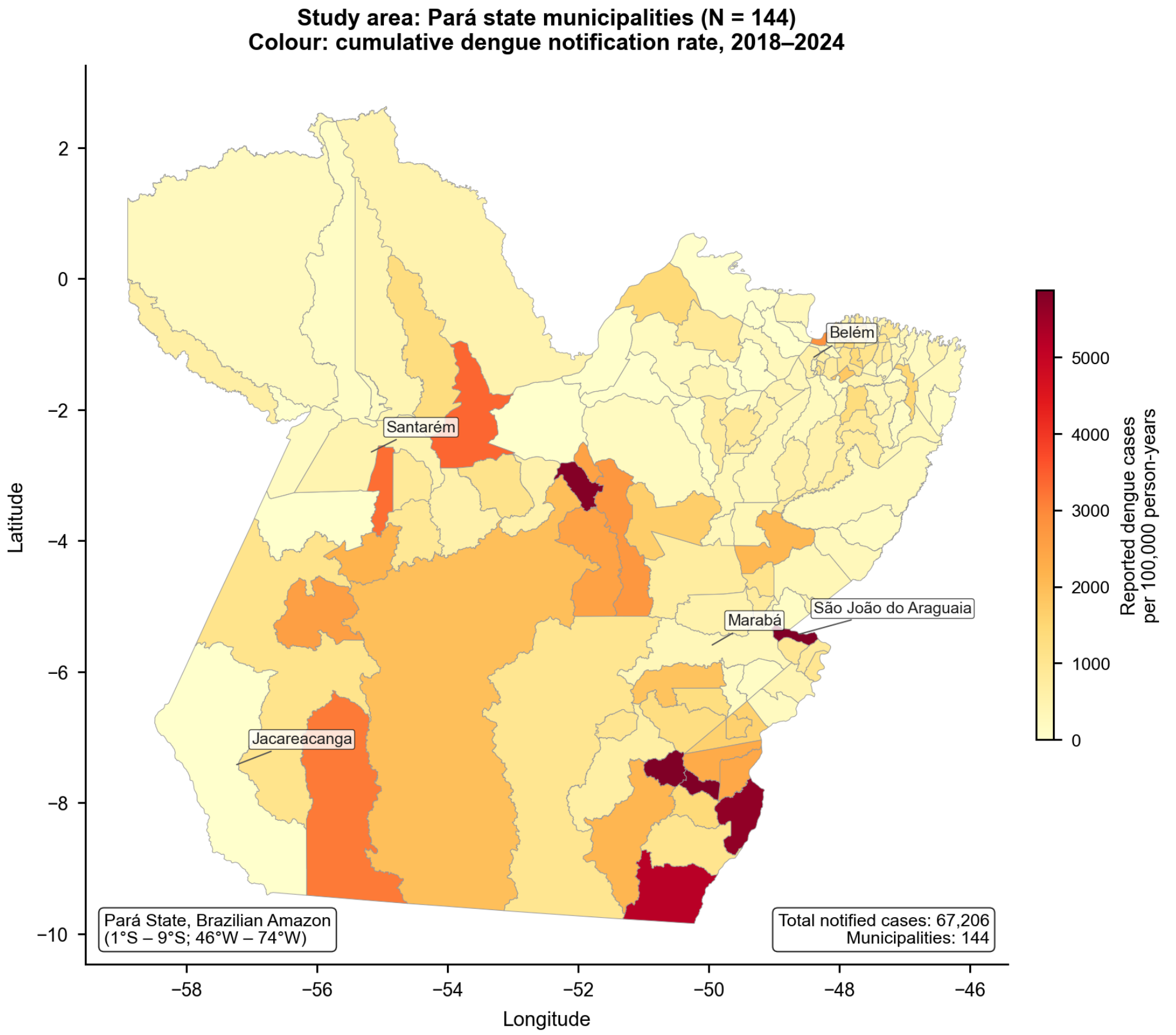

FIGURE S1 Study area: Pará state municipalities (n = 144), Brazilian Amazon.

effects while discouraging overfitting to spurious associations within the 29-dimensional covariate space.

The BYM2 marginal standard deviation σ_s was assigned a prior with concentrated mass on moderate spatial heterogeneity, while remaining sufficiently diffuse to accommodate the large estimated between-municipality variation that characterises Amazonian dengue surveillance. For the ICAR mixing proportion ρ_s, the Jeffreys' prior was used, supporting a balanced formulation between a structured spatial surface and unstructured municipality-level overdispersion, which allows the estimated proportion to be informed primarily by the data. The temporal scale parameters σ_{year} and σ_{sazon} received priors designed to accommodate up to approximately two-fold year-to-year and month-to-month estimated deviations without introducing excessive temporal flexibility. The Negative Binomial dispersion parameter α_{nb} was assigned a prior focusing on the regime of strong overdispersion consistent with heterogeneous surveillance data, while remaining diffuse enough to accommodate less dispersed estimated outcomes if supported by the data.

With all components specified, the model was implemented in PyMC[4] using the No-U-Turn Sampler (NUTS)[5] with the nutpie backend for efficient Hamiltonian Monte Carlo integration. Sampling was conducted across four independent chains, each yielding 2,000 post-warmup estimated draws following 2,000 warmup iterations, for a total of 8,000 post-warmup samples. The target acceptance rate δ_{target} was specified at 0.95, a conservative setting designed to support stable step proposals and mitigate the occurrence of divergent transitions in regions of high posterior curvature.

The spatial adjacency structure required by the BYM2 ICAR component was structured from the 2024 IBGE municipal boundary shapefile[6]. Two municipalities were considered adjacent under a Queen-contiguity rule, formulated as any pair sharing at least one boundary vertex or edge. Applied to the 144 Pará municipalities, this specification supported a connected spatial graph representation with 384 adjacency edges $|\mathcal{E}|$ and an estimated mean of 5.3 neighbours $\bar{d}$ per municipality.

Together, these decisions establish the analytical foundation for the estimated posteriors reported below. The 12,096-cell

panel provides sufficient resolution to support the identification of both aggregate covariate effects and fine-grained spatial heterogeneity. The Negative Binomial likelihood, supported by posterior predictive checking, accommodates the severe overdispersion and high zero fraction inherent in the Pará surveillance data. Weakly informative priors were specified to support data-driven posterior inference while preventing undue prior influence across the estimated covariate space. The NUTS sampler, configured for high acceptance across four independent chains, yielded a posterior sample consistent with computational reliability and appropriate for substantive interpretation.

REFERENCES

1. Instituto do Progresso Social . Metodologia do IPS Brasil 2025 e Base de Dados Municipal. https://ipsbrasil.org.br; 2025.
2. Muñoz-Sabater J, Dutra E, Agustí-Panareda A, et al. ERA5-Land: A state-of-the-art global reanalysis dataset for land applications. *Earth system science data.* 2021;13(9):4349–4383.
3. Hossain S, Islam MM, Hasan MA, et al. Association of climate factors with dengue incidence in Bangladesh, Dhaka City: A count regression approach. *Heliyon.* 2023;9(5).
4. Abril-Pla O, Andreani V, Carroll C, et al. PyMC: a modern, and comprehensive probabilistic programming framework in Python. *PeerJ Computer Science.* 2023;9:e1516.
5. Hoffman MD, Gelman A, others . The No-U-Turn sampler: adaptively setting path lengths in Hamiltonian Monte Carlo.. *J. Mach. Learn. Res.*. 2014;15(1):1593–1623.
6. Instituto Brasileiro de Geografia e Estatística (IBGE) . Malhas Territoriais 2024. https://www.ibge.gov.br/geociencias/organizacao-do-territorio/malhas-territoriais.html; 2024.
